# Elastic stable intramedullary nailing versus Kirschner wire in the treatment of pediatric metaphyseal–diaphyseal junction fractures of the distal radius: a case-control study

**DOI:** 10.1186/s12891-023-07055-9

**Published:** 2023-11-30

**Authors:** Rongchang Wu, Yuwei Wen, Chunhua Wang, Tao Liu, Jiazhi Yu

**Affiliations:** 1 Department of Radiology, Beijing Children’s Hospital, Capital Medical University, National Center for Children’s Health, No. 56, Nalishi Road, Beijing, 100045 China; 2 Department of Orthopaedics, Beijing Children’s Hospital, Capital Medical University, National Center for Children’s Health, No. 56, Nalishi Road, Beijing, 100045 China; 3 Department of Orthopaedics, Children’s Hospital Affiliate to Shandong University (Jinan Children’s Hospital), No.23976 Jishi Road, Shandong Jinan, 250022 China

**Keywords:** Radius fracture, Children, Metaphyseal–diaphyseal junction, Elastic stable intramedullary nail, Kirschner wire

## Abstract

**Background:**

Several methods have been used for the treatment of pediatric distal radius fractures, such as the elastic stable intramedullary nail (ESIN), Kirschner wire (K-wire), and plate, but there has been no consensus about the optimum method. The purpose of this study was to compare ESIN and K-wire techniques used in metaphyseal–diaphyseal junction (MDJ) fractures of the pediatric distal radius.

**Methods:**

The data of patients who were treated at a children’s hospital affiliated with Shandong University between August 2018 and January 2022 were analyzed retrospectively. The children were divided into the ESIN and K-wire groups. Clinical outcomes were measured by the Gartland and Werley scoring system. Variables were analyzed using a statistical approach between the two groups.

**Results:**

The study included 26 patients, of whom 11 were treated with K-wire and 15 with ESIN. At the final follow-up, all of the fractures were healed. There were no differences in terms of age, sex, fracture location, or wrist function score. However, the ESIN was superior to K-wire in operative time, fluoroscopic exposure, and estimated blood loss (EBL).

**Conclusions:**

K-wire and ESIN are both effective methods in the treatment of MDJ fractures of the pediatric distal radius. The use of the ESIN technique represents less EBL, fluoroscopy exposure, and operation time compared with K-wire. We recommend osteosynthesis by ESIN rather than K-wires in patients with MDJ fractures of the distal radius.

**Level of evidence:**

III, a case-control study.

## Background

Distal radius fractures are the most common trauma, representing 30% of all fractures in children [1], and are more common in boys [2]. The peak rate of occurrence is observed at the age of 11.5–14.5 years old [2]; this observation is attributed to increases in bone fragility and physical activity during puberty [3].

Treatment of distal radius fractures remains challenging, with no consensus regarding the best method. Conservative treatment should be the first-line treatment for forearm fractures, primarily because of the greater potential for growth in these patients and thus remodeling [4]. However, loss of fracture reduction and re-displacement is a major complication, and the reported incidence ranged from 21 to 47% [5, 6]. Surgical treatment should be considered for unstable and displaced distal radius fractures [7–9].

The metaphyseal–diaphyseal junction (MDJ) is a noteworthy area located at the distal radius, which was previously defined as the area encompassing the distal radius and ulnar physis minus the square encompassing the distal radial physis [10]. There are several treatment methods for MDJ fractures, such as Kirschner wires (K-wires), plates, external fixators, and elastic stable intramedullary nails (ESIN) [10–15], and there is no consensus regarding superiority. Plate fixation is the most invasive modality and has a wide range of complications. The large wounds resulting from plate fixation are inconsistent with the concept of minimally invasive surgery. External fixators are also associated with several complications, such as infections, the risk of self-harm, delayed fracture union, and refractures after implant removal.

ESIN and K-wires are two commonly used treatments for distal radius fractures in children. However, few studies have compared the outcomes of these two methods. The objective of this study was to retrospectively compare the therapeutic effects of K-wire fixation and ESIN for MDJ fractures of the distal radius. We hypothesized that ESIN is the optimal method for treating MDJ fractures of the pediatric distal radius.

## Methods

### Demographics

**Fig. 1:**
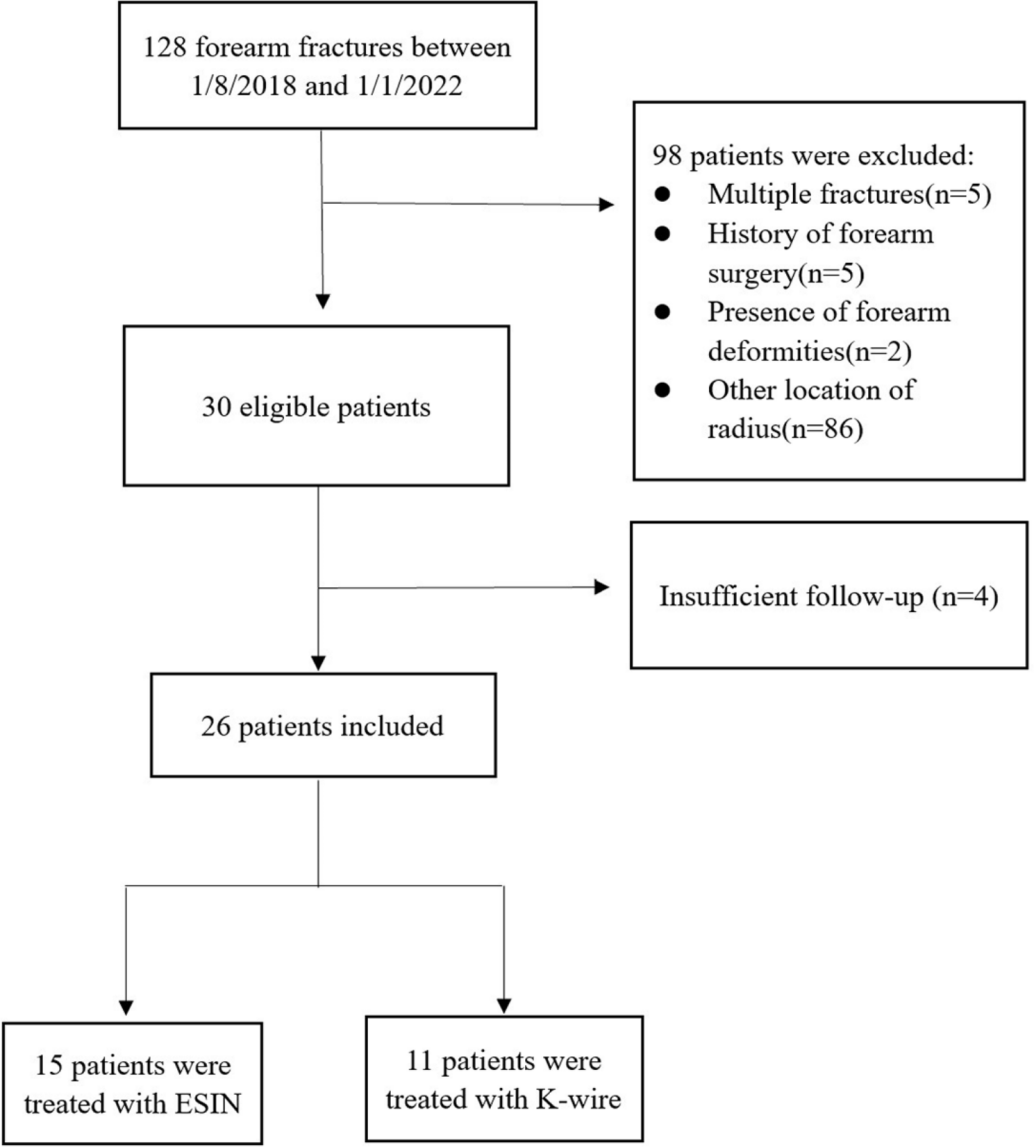
Flowchart of the study population

The data of children with forearm fractures in Children’s Hospital Affiliate to Shandong University (Jinan Children’s Hospital) between August 2018 and January 2022 were analyzed. The inclusion criteria were as follows: MDJ fractures and those combined with distal ulnar fractures, failed conservative treatment or unstable fractures, and the presence of open epiphyseal plates. The exclusion criteria were multiple fractures, pathological fractures, a history of forearm surgery, and the presence of forearm deformities. Accordingly, 26 children with sports injuries as the main cause of their fractures were included (Fig. 1).

All patients with fracture lines located less than 2 cm away from the physis underwent K-wire fixation. Patients with fracture lines located more than 2 cm away from the physis were treated with either K-wire or ESIN.

The following demographic data were collected: operation time, estimated blood loss (EBL), fluoroscopy time, length of stay, and postoperative complications (needle tract infection, postoperative fracture displacement, nonunion, delayed union, malunion, wrist stiffness, and nerve injury). At 12 months postoperatively, the Gartland and Werley wrist function scoring system was used to evaluate wrist function based on residual deformity, subjective evaluation, objective evaluation, and postoperative complications. The scores of 0–2, 3–8, 9–20, and ≥ 21 were considered excellent, good, fair, and poor, respectively [16].

### Operative technique

The patients were anesthetized using an intravenous infusion. If manipulative reduction of the fracture failed, small incision-assisted prying was performed; satisfactory fracture reduction was confirmed using C-arm fluoroscopy. In the K-wire group, appropriate K-wires were selected based on the patients’ age. For bicortical fixation, two K-wires were inserted through the distal radius while ensuring that they did not cross the epiphyseal plate. In the ESIN group, a 5-mm skin incision was made on the posteromedial side of the distal radius, which was separated from the bone cortex. The elastic nail diameter was two-thirds of the narrowest part of the radial marrow cavity; the nails were pre-curved into an L-shape and placed near the radial neck. Fluoroscopy confirmed fracture reduction and satisfactory positioning of the nail. The tail of the nail was cut and buried under the skin. Generally, if a concurrent distal ulnar fracture is present, K-wire fixation, ESIN, or conservative treatment can be performed.

In all patients, the affected forearm was immobilized in a neutral position for 4 weeks using a long cast. The cast and K-wires were removed 4 weeks after surgery, and functional wrist exercises were initiated. The ESIN was removed 4–8 months postoperatively.

### Statistical analysis

Statistical analyses were performed by the SPSS 22. The Kolmogorov–Smirnov test was performed to inspect the normality of the measurement data. Normally distributed variables were analyzed using a t-test. Non-normally distributed data were analyzed by non-parametric tests. The count data were analyzed by the chi-square test. The level of significance was set at p < 0.05.

## Results

The ESIN group comprised 15 patients. The mean age was 7.7 ± 2.0 years, with ten males and five females. Radial fractures were combined with ulnar fractures in 8 patients. The K-wire group comprised 11 patients (nine males and two females; the mean age was 6.4 ± 1.6 years). Radial fractures were combined with ulnar fractures in 7 patients.

**Fig. 2:**
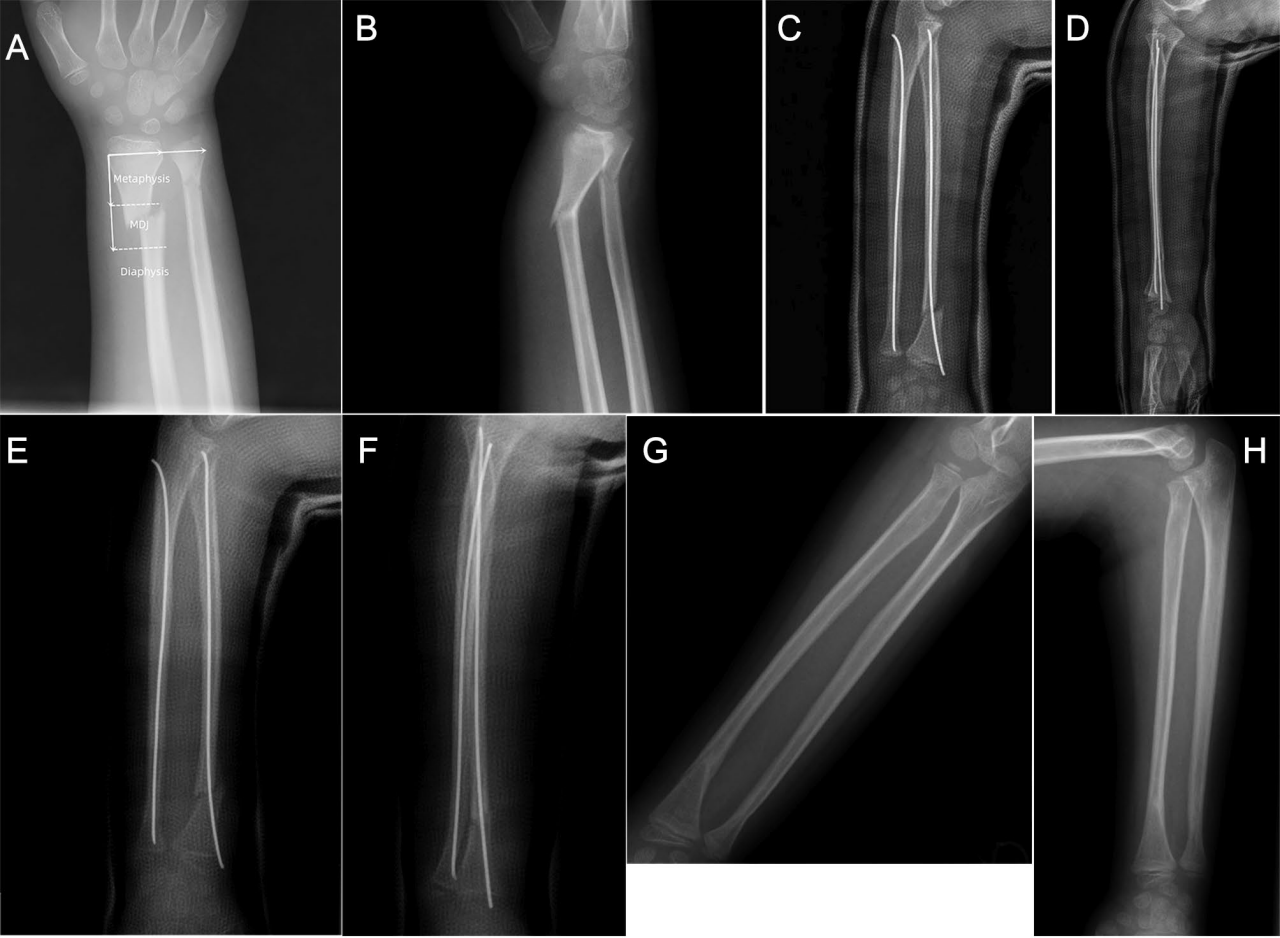
(**A**) Definition of an MDJ fracture of the distal radius. A 9-year-old boy presented with a right distal radius and ulna. (**A**, **B**): Radial fractures are observed in the MDJ. (**C**, **D**): Closed reduction and ESIN are performed. (**E**, **F**): Radiographic examination performed 1 month after the surgery reveals radiographic union. (**G**, **H**): The elastic nail was removed 4 months after the surgery. Final follow-up findings show fracture healing and no deformities. MDJ: metaphyseal-diaphyseal junction; ESIN: elastic stable intramedullary nailing

All patients underwent a closed reduction without conversion to an open reduction. The follow-up time was 14 months (range 12–24 months). In all patients, fracture union was achieved at the final follow-up; no cases of pin-site infection, nerve injury, wrist stiffness, or postoperative re-displacement were noted. The wrist score was excellent in 13 cases and good in 2 cases in the ESIN group, and excellent in 8 and good in 3 patients in the K-wire group. The rates of excellent and good scores were 100% in both groups (Fig. 2).

Imaging performed 4 weeks postoperatively revealed fracture healing in all children. The cast and K-wire were removed, and functional exercises were initiated; there was no delayed union or nonunion. Two patients in the ESIN group had residual angular deformities of 9° and 8° at the last follow-up; however, these had no impact on their wrist function. The time of implant removal was 5.6 months (4–8 months).

There was no difference in terms of age, sex, fracture side, ulnar fracture incidence, length of hospital stays, or wrist function scores. However, ESIN was superior to K-wire due to its shorter operation and fluoroscopy times and lower EBL (Table 1).

**Table 1:** Clinical data and statistical analysis results

Group Project				ESIN	K-wire	t/z /x^2^	p
Sex		Boys		10	9	0.74	0.66
		Girls		5	2		
Side		Lift		9	7	0.04	1.00
		Right		6	4		
Fracture		Radius		7	4	0.28	0.70
		Radius + ulnar		8	7		
Age(y)				7.7 ± 2.0	6.4 ± 1.6	-1.78	0.88
Operation time(min)		Radius		35.8 ± 11.88	65.22 ± 20.20	3.92	0.01
		Radius + ulnar		44.33 ± 14.92	79.50 ± 20.51	2.69	0.04
Intraoperative fluoroscopy (t)				6.53 ± 2.42	10.27 ± 3.35	3.31	0.03
Estimated blood loss (ml)				3.5 ± 2.0	5.6 ± 2.8	2.14	0.04
Length of stay(d)				4(3,5)	4(3,4)	-0.85	0.40
Score		Excellent		13	8	0.80	0.62
		Good		2	3		

## Discussion

The optimal treatment for pediatric radius fractures was a simple, less traumatic procedure. In the current study, we found that ESIN was associated with lower EBL and shorter operative and fluoroscopy times compared with K-wire, while both techniques yielded similar wrist function.

Management of pediatric distal radius fractures is affected by several variables, including age, fracture pattern, and epiphyseal plate involvement; the optimal treatment method for these injuries remains unclear. Vito et al. hypothesized that conservative treatment is the optimal technique for fractures displaced by less than 50% [17]. However, a complete fracture may easily be displaced after a closed reduction. Cast index is a much more reliable parameter for evaluating reduction failure and conservative treatment of forearm pediatric fractures. A cast index > 0.84 indicates a high risk for conservative treatment failure [18]. Moreover, multifactor analysis should be considered. Factors impacting secondary displacement include age, radial translation, radioulnar fracture, fracture distance from the physis, and reduction quality [17, 19, 20].

Internal fixation is recommended when malformations cannot be fully corrected during manipulation. The American Academy of Orthopaedic Surgeons recommends moderate-strength surgical fixation over cast fixation for distal radius fractures with unsatisfactory reduction [8]. However, internal fixation options are often demanding or technically impractical. The MDJ intramedullary diameter becomes narrower, and K-wires are difficult to use when stabilizing MDJ fractures [21]. ESIN is the “gold standard” treatment for pediatric radioulnar diaphysis fractures [22], but is not considered appropriate for treating MDJ fractures due to difficult insertion and insufficient stability. Therefore, further cast immobilization is recommended. The Epibloc system (ES) allows a rapid functional recovery without the use of further cast immobilization. Passiatore et al. [23] found that the ES represents faster functional recovery and rarely requires postoperative physiotherapy compared with cast immobilization. De Vitis et al. [24] reported that ES applied with a minimal technical variation is safe and effective in treating distal ulna and radius fractures, with minimal requirements for post-surgical rehabilitation. Du et al. attempted to treat MDJ fractures using anterograde ESIN [13]. Anterograde ESIN cannot achieve three-point fixation and is not superior to traditional fixation. In addition, anterograde ESIN may damage the radial nerves, resulting in radial paralysis that has been observed to regress spontaneously. Retrograde ESIN remains the conventional treatment for distal radius fractures. Furthermore, maximal distal insertion and nail procurement may achieve stability in MDJ fractures [25]. Joulié et al. report that 16 patients were treated with ESIN and achieved satisfactory results [15].

In the current study, K-wire fixation reliably leads to longer operative times than ESIN. ESIN surgery was shorter than K-wire surgery by nearly 30 min. MDJ fractures are located relatively far from the metaphysis; this makes sufficient bicortical fixation with K-wires technically demanding and results in a steep implant angle. Therefore, repeated adjustment of the K-wire’s position prolongs the operative time. A longer operative time also predicts more intraoperative fluoroscopy. ESIN fluoroscopy (6.5 min) took nearly half as long as K-wire fixation (10.3 min). Li et al. reported a fluoroscopy time of 11.6 min for K-wire fixation [10]; although comparative literature for ESIN and K-wire is lacking, this mean fluoroscopy time is longer than ours.

Plate fixation for MDJ fractures enhances anatomical reduction, completely corrects malrotation, and restores the arch shape of the radius [26]. However, open reduction and plate fixation require extensive dissection, which adversely affects fracture healing. Patients experience a longer recovery period and a delayed return to sports activities following open reduction maneuvers [10]. Limited rotational function of the forearm secondary to damaged soft tissue structure of the forearm has been reported after plate fixation [27].

Children have sufficient remodeling potential; therefore, the purpose of the procedure is to achieve stable fixation rather than anatomical reduction. In our study, the ESIN was bent in an L-shaped manner to obtain sufficient stability. The apex of the L-shape is located distal to the fracture line, such that it utilizes the recovery force to maintain fracture reduction. The insertion point was selected according to the fracture–displacement model. MDJ fractures with anterior displacement have been suggested to be appropriate for retrograde posteromedial ESIN [28]. If the fracture location is not distal, ascending posteromedial nailing should be considered. This is because the nail’s elastic force is beneficial for maintaining reduction, given its posterior entry point on the distal radius [15]. Anterolateral insertion points are selected for dorsal angulation. If the extensor tendons are cautiously separated using dissection forceps, their transfixion is prevented. Additional casting is necessary for radial MDJ fractures to compensate for the instability associated with ESIN. In this study, all the children treated using the above-mentioned comprehensive measures showed closed reductions, no re-displacement, and excellent or good wrist function.

This study has the following limitations. This was a retrospective case–control study, and a selection bias may exist in the way that the patients were chosen for the procedures. The smaller sample size resulted in lower confidence. Thus, we believe that future prospective studies with larger sample sizes are carried out to warrant our findings. The follow-up time is shorter, and future studies need to extend the follow-up time.

## Conclusions

K-wire and ESIN are both effective methods in the treatment of MDJ fractures of the pediatric distal radius. However, ESIN was superior to K-wire fixation for the treatment of radial MDJ fractures in children. Compared with K-wire fixation, ESIN offered shorter operation and fluoroscopy times and less EBL. The radiographic outcomes and wrist joint function after ESIN were favorable, and there was no re-displacement, nonunion, or delayed union.

## Data Availability

The datasets used and/or analyzed during the current study are available from the corresponding author upon reasonable request.

## List of abbreviations

ESIN: Elastic stable intramedullary nailing
K-wire: Kirschner wire
MDJ: Metaphyseal–diaphyseal junction
EBL: Estimated blood loss
ES: Epibloc system
